# Foot orthoses in the treatment of symptomatic midfoot osteoarthritis using clinical and biomechanical outcomes: a randomised feasibility study

**DOI:** 10.1007/s10067-015-2946-6

**Published:** 2015-04-28

**Authors:** Jill Halstead, Graham J. Chapman, Janine C. Gray, Andrew J. Grainger, Sarah Brown, Richard A. Wilkins, Edward Roddy, Philip S. Helliwell, Anne-Maree Keenan, Anthony C. Redmond

**Affiliations:** Section of Clinical Biomechanics and Physical Medicine, Leeds Institute of Rheumatic and Musculoskeletal Medicine, University of Leeds, 2nd Floor, Chapel Allerton Hospital, Harehills Lane, Leeds, LS7 4SA UK; Leeds Institute of Clinical Trials Research, University of Leeds, Leeds, UK; Leeds NIHR Musculoskeletal Biomedical Research Unit, Leeds Teaching Hospitals Trust, Leeds, UK; Department of Musculoskeletal Radiology, Leeds Teaching Hospitals Trust, Leeds, UK; Research Institute for Primary Care and Health Sciences, Keele University, Keele, UK; Arthritis Research UK Experimental Arthritis Centre, Leeds, UK; Arthritis Research UK Centre for Sports, Exercise and Osteoarthritis, Nottingham, Oxford, Loughborough, Leeds, UK

**Keywords:** Feasibility, Foot, Functional foot orthoses, Gait, Midfoot osteoarthritis, Osteoarthritis, Randomised trial

## Abstract

This randomised feasibility study aimed to examine the clinical and biomechanical effects of functional foot orthoses (FFOs) in the treatment of midfoot osteoarthritis (OA) and the feasibility of conducting a full randomised controlled trial. Participants with painful, radiographically confirmed midfoot OA were recruited and randomised to receive either FFOs or a sham control orthosis. Feasibility measures included recruitment and attrition rates, practicality of blinding and adherence rates. Clinical outcome measures were: change from baseline to 12 weeks for severity of pain (numerical rating scale), foot function (Manchester Foot Pain and Disability Index) and patient global impression of change scale. To investigate the biomechanical effect of foot orthoses, in-shoe foot kinematics and plantar pressures were evaluated at 12 weeks. Of the 119 participants screened, 37 were randomised and 33 completed the study (FFO = 18, sham = 15). Compliance with foot orthoses and blinding of the intervention was achieved in three quarters of the group. Both groups reported improvements in pain, function and global impression of change; the FFO group reporting greater improvements compared to the sham group. The biomechanical outcomes indicated the FFO group inverted the hindfoot and increased midfoot maximum plantar force compared to the sham group. The present findings suggest FFOs worn over 12 weeks may provide detectable clinical and biomechanical benefits compared to sham orthoses. This feasibility study provides useful clinical, biomechanical and statistical information for the design and implementation of a definitive randomised controlled trial to evaluate the effectiveness of FFOs in treating painful midfoot OA.

**Electronic supplementary material:**

The online version of this article (doi:10.1007/s10067-015-2946-6) contains supplementary material, which is available to authorized users.

## Introduction

Osteoarthritis (OA) is a common cause of joint pain and disability [1–3]. Previous population studies have suggested that the most common site for OA in the foot is the first metatarsophalangeal joint (prevalence of approximately 22 %), with midfoot OA reported as being relatively uncommon (prevalence of 3.8 %) [4, 5]. Using a recently developed foot atlas, which includes the first metatarsophalangeal joint, and medial midfoot joints (first metarso-cuneiform joint, second metatarso-cuneiform joint, navicular-cueniform joint and talo-navicular joint), two large population studies have demonstrated that medial midfoot OA is more prevalent than previously reported [6, 7]. The prevalence of medial midfoot OA in an older population was 88 % (compared to 95 % in the metatarsophalangeal joint); while in a younger community study, the prevalence of medial midfoot OA was 7.8 % (compared to 6.8 % in the metatarsophalangeal joint), contributing to the prevalence of disabling foot pain [8]. Compared to the hip and knee OA, there are few studies investigating the potential interventions for midfoot OA.

Midfoot OA is associated with movement impairment, structural deformity and increased foot pressures [9–11]. Modification of these factors, via functional foot orthoses (FFOs), provides a possible mechanism for biomechanically based clinical treatments. Two previous clinical midfoot OA studies have demonstrated improvements in pain and function following the use of FFOs over 4 weeks [12] and 6 months [13], although neither employed a randomised placebo or sham control. NICE guidelines [14] recommend that foot orthoses should be considered as an adjunct therapy for OA despite the lack of quality randomised controlled trials (RCTs) as they carry minimal risk. This study was undertaken under the auspices of the Arthritis Research UK Clinical Studies Group for Osteoarthritis and Crystal Diseases to examine the feasibility of conducting a definitive RCT (Orthoses in Foot Function and Loading in OA Disease: OFFLOAD). The aim of this feasibility study was to determine whether functional foot orthoses have the potential to help painful and disabling midfoot osteoarthritis. This was addressed through three objectives: (1) to explore the key methodological issues for a future RCT, (2) to establish whether FFOs improve midfoot OA-related pain and function over 12 weeks and (3) to explore FFOs alter biomechanical outcomes (hindfoot kinematics and midfoot forces) compared to a sham device.

## Materials and methods

### Study design

**Fig. 1:**
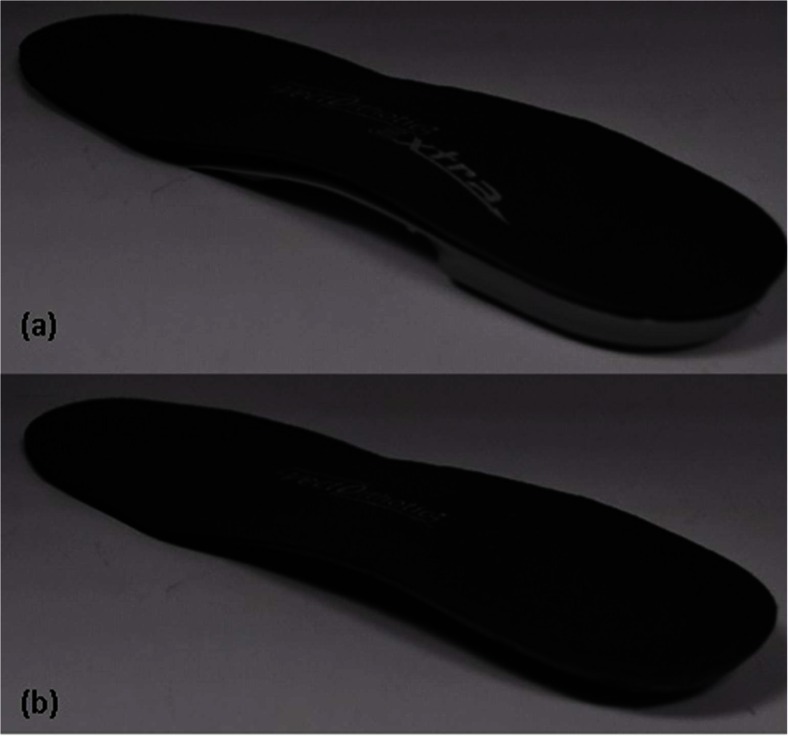
Diagram illustrating the posterior-medial view of the interventions **a** functional foot orthoses and **b** sham

The study was a double-blind, two-arm parallel group randomised controlled feasibility study. Participants were randomised at baseline to receive either a pair of ‘active’ FFOs (see Fig. 1a) in the intervention group or sham orthoses (see Fig. 1b) in the control group (please see Supplementary file for details). The term ‘active FFOs’ in the context of this study relates to three functional features; a contoured semi-rigid shell, a stabilising cupped heel and the use of heel wedging to influence foot position. Randomisation was conducted on a 1:1 basis, with no stratification by a blinded member of the study team (RAW), according to a random number algorithm contained in pre-sealed envelopes [15]. The study was designed to recruit 20 participants in each group [16], with a follow-up period of 12 weeks to allow a reasonable clinical assessment and feasibility of compliance and attrition to be evaluated.

### Participants

Participants were recruited from a community musculoskeletal service. Potential participants were verbally and clinically screened to ensure they met inclusion/exclusion criteria. Patient-reported medical history and medications were recorded by the main researcher (JH), and physician-confirmed (from GP medical record summary) concomitant OA in the body was recorded on a manikin by the patient. Participants were included if they were ≥18 years of age, reported foot pain for ≥3 months, located the foot pain within the midfoot region by drawing the location on a foot pain manikin in predetermined dorsal and medial regions of the foot [17] (© The University of Manchester 2000. All rights reserved) and reported midfoot pain occurring with or worsening immediately following weight-bearing activities. All participants had radiographic midfoot OA verified on weight-bearing radiographs by a musculoskeletal radiologist (AG) using predetermined criteria recommended in the La Trobe University Atlas of Foot Osteoarthritis [6]. Osteoarthritis-related foot pain was defined by a score >2/10 on an 11-point numerical rating scale (NRS) for average foot pain the last week and at least one criteria of the foot function impairment reported on most days (Manchester Foot Pain and Disability Index [MFPDI] [18]). Exclusion criteria were contraindications to radiographs or gait analysis; history of suspected or confirmed inflammatory joint disease, neuropathy or stress fractures; history of lower limb bone and joint surgery in the last 12 months; or existing use of over-the-counter or prescribed foot orthoses.

To avoid breaching assumptions of statistical independence in bilateral limb studies, one limb per participant was included in the analyses [19]: if participants reported midfoot pain in both feet, the most painful foot was used as the study limb. If midfoot pain was equal in both feet, the dominant foot was included (defined by first step initiation). Bradford NHS research ethics committee approval was obtained (reference: 12/YH/0093), and all participants provided written informed consent prior to commencing the study according to the Declaration of Helsinki.

### Intervention

In the FFO group, participants received a pair of firm semi-rigid FFOs (VectOrthotic® Healthy Step [Sensograph] Ltd), which contoured into the arch and supported the midfoot with the aim of controlling joint motion. Functional foot orthoses were prescribed as per standard clinical practice and customised to each participant by an experienced clinical podiatrist (JH) (see Fig. 1a and Supplementary file for details).

The sham group received orthoses that mimicked the appearance of the active intervention but without firm midfoot support and heel wedging (see Fig. 1b and Supplementary file). It was hypothesised that the sham intervention had some cushioning properties but none of the significant mechanical characteristics of the active FFO (see Supplementary file for details) and could be deemed a sham [20]. A footwear advice leaflet was provided to all participants providing fitting and contact information.

### Intervention blinding

In the patient information sheet, the two types of foot orthoses were presented; either joint controlling or cushioning. It was not implied which intervention was superior; only that a fair evaluation of two types of orthoses were being tested. Participants were blind to the treatment allocation in order to limit assessment and expectation bias, and every attempt was made to maintain the blind. A single researcher (JH) was responsible for the provision of orthoses and clinical care but was not involved in the acquisition of patient-reported outcome measures (PROMs) at follow-up. The preservation of blinding was formally examined by interview at the end of the study.

### Data capture and outcome measures

#### Patient-reported outcome measures

All PROMs were validated and entered by a second researcher (RAW) who remained blinded to treatment allocation. The clinical outcomes were change in midfoot pain and foot disability scores from baseline to 12 weeks, chosen according to current research and recommendations for chronic pain trials (IMMPACT guidelines [21]):

1. A number of foot pain questions were used to examine clinical responsiveness (for a subsequent full RCT), each assessed using an 11-point numeric rating scale scored from ‘no pain’ to ‘pain as bad as you can imagine’: The anchor questions were (i) worst foot pain in the last 24 h, (ii) average foot pain in the last 24 h, (iii) average foot pain in the last week, (iv) average foot pain in the last month, and (v) average foot pain while walking in the last week [21].
2. Patient Global Impression of Change (PGIC). Participants rated their perception of clinical improvements in foot pain and foot pain when walking, using a 7-point Likert scale [22].
3. Foot function, measured using the function subscale of the MFPDI [18].

#### Treatment adherence

Adherence was measured daily using a self-reported diary to record the number of hours wear per week over the 12 weeks. Adherence was based on the mean number of hours per week, per participant over the 12 weeks to examine between-group differences.

#### Examination of intervention blinding

The success of the blinding was investigated by asking participants at the end of the study to identify which type of intervention were you provided (1) ‘controlling orthoses’, (2) ‘cushioning orthoses’ or (3) ‘do not know’. For analysis purposes, participant answers were categorised into three participant responses as correctly identified, incorrectly identified or unknown; the proportions for each response were calculated as a percentage.

#### Biomechanical outcome measures

To investigate potential biomechanical effects of the orthoses, in-shoe plantar pressures and foot kinematics were obtained. In order to avoid data mining, a limited number of variables were chosen a priori and explored in this study.

The force redistribution through the midfoot was captured using the Pedar® in-shoe system (Novel GmbH, Munich) acquired at 50 Hz. During the study, the allocated orthoses were worn in the participant’s own shoes, but to minimise the confounding effect of different shoe types and to accommodate both the randomised intervention and the measuring Pedar insole, a standardised shoe was worn by each participant during the laboratory acquisition. Each participant underwent gait analysis in a standard shoe-only condition and wearing the standard shoe plus their randomised intervention in a prespecified random order. The standard shoe consisted of an open-webbed netting upper into which slits were cut allowing for visualisation of the markers and acquisition of 3D foot kinematics (see below). The shoe also had a flat rubber sole with no plantar contouring. After 5 min of acclimatisation, participants walked four times across a level 10-m gait laboratory walkway at a self-selected speed. Measures were conducted in a prespecified random sequence and between 12 and 16 mid-pass steps were obtained under each experimental condition.

Force data were derived using the Novel-win program (version 0.8 Novel Win GmbH, Munich) with a Novel percent mask dividing the study foot into three regions: hindfoot (31 %), midfoot (33 %) and forefoot (36 %). For each participant, the mean difference (intervention condition minus shoe-only condition) in midfoot maximum force (% of body weight [BW]) was calculated.

Multi-segment foot kinematics were captured using 9 mm reflective markers attached to the skin in accordance with the Oxford multi-segment foot model [23]. Kinematic data were captured at 200 Hz using an eight camera motion capture system (Vicon MX, Oxford Metrics, UK), integrated with a force plate (Bertec Corporation, USA) capturing at 1000 Hz. Each participant underwent gait analysis in the standard shoe-only condition and the standard shoe plus their randomised intervention in a prespecified random order. A static trial was captured in a neutral reference position (Foot Posture Index score = 0 [24]). For both experimental conditions, each participant completed six walking trials at a self-selected speed.

Kinematic data were exported to Visual 3D (C-Motion Inc., Rockville, MD, USA) for further analysis. Kinematic data were filtered using a low-pass fourth-order Butterworth filter with a cut-off frequency of 6 Hz and normalised to stance phase centiles to enable averaging across trials and conditions. Peak angular frontal plane motion of the hindfoot with respect to the tibia was selected as the predefined variable of choice to determine whether the FFO demonstrated greater constraint on the hindfoot than the sham. At the follow-up appointment, the mean difference between the orthoses and shoe-only condition was calculated for each participant.

### Statistical analysis

Statistical analysis was planned and undertaken by the Leeds Clinical Trials Research Unit by statisticians blinded to the intervention allocation (SB and AD). Descriptive assessment indicated that data was sufficiently normally distributed to report mean, standard deviation (SD) and 95 % confidence intervals (CI). Clinical outcome measures and biomechanical outcomes, reported as mean differences between groups (FFO minus sham groups) with associated 95 % CI, were used to explore the effectiveness of FFOs on pain and function. The PGIC Likert scale was collapsed to summarise the proportion indicating clinical improvement in each group at 6 and 12 weeks. Descriptive statistics were used to describe the feasibility outcomes relating to the key methodological issues of this feasibility study. The data was assessed and summarsied using SAS version 9.2. (SAS Inst Inc, NC, USA).

Pre-specified minimally important differences were identified prior to study commencement. These included:

I. Improvements from baseline to 12 weeks in foot pain using multiple NRS anchors, with the mean difference between the two treatment groups greater than 1.5 points [25, 26].
II. Improvements from baseline to 12 weeks of the functional subscale of the MFPDI, with a mean difference between treatment groups of three points or more [27].
III. At 12 weeks, the mean between-group reduction in peak hindfoot eversion for shoe-only minus shoe-plus randomised intervention of a mean of 2.1° or greater [28].
IV. At 12 weeks, the mean between-group difference for shoe-only minus shoe-plus allocated intervention increasing midfoot force by a mean of 21 % or greater [29].
V. The inert sham altering mean peak hindfoot inversion by less than 2° and changing midfoot force by less than 20 %.
VI. Adequate adherence with allocated orthoses set at a weekly average of 21 h wear for 80 % of participants in the group.

In addition, treatment blinding, recruitment and attrition rates were evaluated descriptively to explore the feasibility of recruitment, retention and success of blinding participants in a subsequent RCT.

## Results

### Feasibility outcome measures

#### Recruitment

**Fig. 2:**
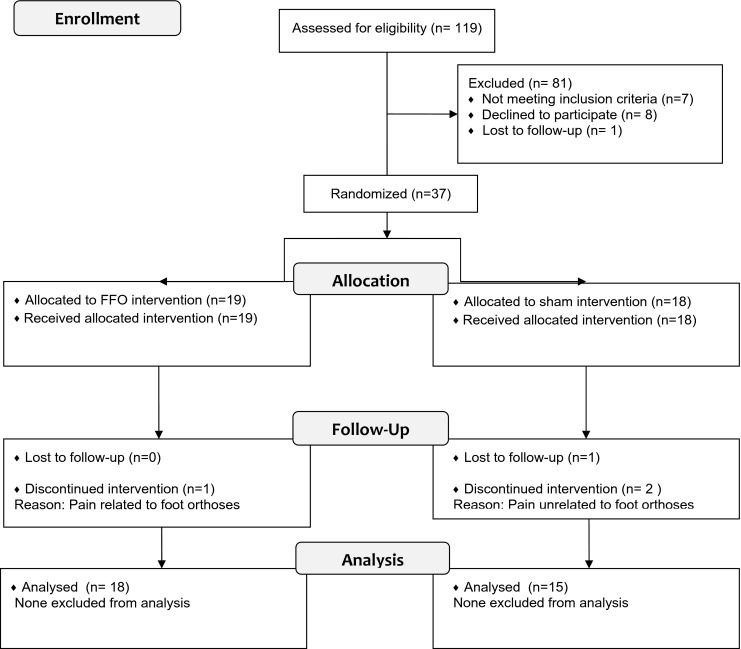
Flow chart of participants through the study (CONSORT 2010 statement)

Over 8 months, 119 potential participants were screened, of whom 46 were eligible. Of these 46 participants, eight declined and one was lost to follow-up, resulting in 37 (31 % of screened participants, 95 % CI 23 % to 40 %) being randomised. Nineteen participants were randomised to the FFO group and 18 to the sham control group (see Fig. 2).

At follow-up, four participants did not complete the study (11 % attrition rate). In the FFO group, one participant was withdrawn due to escalating back pain and burning pains in her feet. In the sham group, two participants were unable to complete the study due to sudden unrelated back pain and one participant was lost to follow-up.

#### Participant demographics and clinical characteristics

Participants in the two groups were well matched for age, although there was a higher mean BMI in the FFO group and a slightly larger proportion of female participants compared to the sham group (see Table 1). The study cohort presented with a number of common comorbidities with the most common being hypertension and hypercholesterolemia (mean 2, range 0–9). In addition, most of the participants reported concomitant OA in the proximal joints; medial knee OA was the most common location (70.3 %).

**Table 1:** Baseline demographics and clinical characteristics of participants

	Total group (*n* = 37)	Functional foot orthoses (*n* = 19)	Sham intervention (*n* = 18)
Age (years)	58.4 (11.6)	60.5 (10.4)	56.2 (12.6)
Gender (female)	26 (70.3 %)	15 (78.9 %)	11 (61.1 %)
Body mass index (kg/m^2^)	29.5 (4.5)	31.2 (4.5)	27.7 (3.9)
Right foot affected (proportion)	20 (54.1 %)	11 (57.9 %)	9 (50.0 %)

Values are reported as mean (SD) unless otherwise stated

Using a foot manikin [18], all participants localised their foot pain to the dorsal midfoot region, and six participants further localised their pain in the medial arch region. The type of pain was mostly described as aching or dull, and around half of participants (57 %) described a pattern of intermittent sharp pain associated with weight-bearing activity. The median number of OA affected midfoot joints was two. The most frequent site was the cuneiform-second metatarsal joint (73 %), followed by the naviculo-medial cuneiform joint (51 %), the cuneiform-first metatarsal joint (46 %) and the talo-navicular joint (24 %).

#### Adherence with treatment

Participants wore their allocated orthoses a mean of 39 h per week, and 85 % wore their allocated orthoses for >21 h. The sham group wore their intervention an average of 18 h/week longer than the FFO group (see Table 2). At 12 weeks, 93 % of participants in the sham group reached the prespecified mean of at least 21 h/week adherence compared to 78 % of the FFO group.

**Table 2:** Differences in clinical and feasibility outcome measures. Patient-reported outcomes are reported both within group to describe change over time and between group (FFO vs sham) for differences at the primary endpoint (12 weeks). Biomechanical and feasibility outcomes are reported as between group (FFO vs sham) only

Outcome measures								
		Functional foot orthoses group (FFO)			Sham intervention group (sham)			Mean difference FFO-sham (95 % CI)
Type of outcome measure		Baseline (*n* = 19)	12 weeks (*n* = 18)	Difference (12 weeks − baseline)^c^	Baseline (*n* = 19)	12 weeks (*n* = 18)	Difference (12 weeks − baseline)^c^	
Patient reported	Pain in last 24 h (NRS)	5.6 (2.0)	4.5 (2.0)	−1.1 (2.5)	4.7 (2.4)	4.6 (2.8)	0.3 (3.4)	−1.4 (−3.5 to 0.7)
	MFPDI function	10.5 (4.1)	6.5 (4.7)	−3.6 (3.8)	9.8 (5.3)	8.4 (5.2)	−2.2 (4.1)	−1.4 (−4.1 to 1.4)
	PGIC (%)		83.4			46.6		36.8 (6.1 to 67.2)
Biomechanical	Max Midfoot Force (% BW)^b^		10.7 (6.6)			4.4 (6.3)		6.3 (1.7 to 10.9)
	Peak hindfoot angle (SD)^a^		0.7° (1.6°)			−0.3° (2.5°)		1.04° (−0.5° to 2.6°)
Feasibility	Mean adherence (hours/week)		30.9			48.9		
	Participant adherence ≥21 h/week		14/18 (78 %)			14/15 (93 %)		
	Blinding maintained		14/18 (78 %)			11/15 (73 %)		

Values are reported as mean (SD) unless otherwise stated

*NRS* numeric rating scale, *MFPDI function* Manchester foot pain and disability index—functional subscale, *PGIC* patient global impression of change

^a^Negative values correspond with hindfoot eversion

^b^Calculated as maximum force with intervention − maximum force without intervention

^c^Difference between outcomes at baseline and 12 weeks with missing values removed

#### Treatment blinding

Overall, 17 participants who completed the trial reported being unsure of their treatment allocation (FFO *n* = 12/18; sham *n* = 5/15) (see Table 2). The allocated intervention was incorrectly identified by eight participants (FFO, *n* = 2/18; sham, *n* = 6/15) and correctly identified by eight (four in each group). Combining the number who could not identify the type of intervention (*n* = 17) with those who incorrectly identified the intervention (*n* = 8), blinding was successfully achieved in most of the participants (*n* = 25/33) with only minor differences between the groups (FFO = 14/18, sham = 11/15).

#### Clinical outcome measures

The FFO group demonstrated a greater reduction in mean worst-rated foot pain in the previous 24 h (−1.4, 95 % CI −3.5 to 0.7) and a greater reduction in the functional subscale of the MFPDI (−1.4, 95 % CI −4.1 to 1.4) compared to the sham group (see Table 2). Both groups reported improvements in foot pain after 12 weeks (proportion of participants reporting improvement using PGIC scale); FFO = 83.4 %; sham = 46.6 %, demonstrating a between-group mean difference of 36.8 % (95 % CI 6.1 to 67.2). The results of the additional anchoring pain questions are shown in Table 3.

**Table 3:** Differences in numeric rating scale (NRS) pain outcomes for different anchoring questions within group and between treatment groups from baseline and 12-week follow-up

Clinical outcome measures							
	Functional foot orthoses group (FFO)			Sham intervention group (sham)			Mean difference of FFO-sham (95 % CI)
	Baseline (*n* = 19)	12 weeks (*n* = 18)	Difference (12 weeks − baseline)^a^	Baseline (*n* = 19)	12 weeks (*n* = 18)	Difference (12 weeks − baseline)^a^	
Pain at its worst in last 24 h	5.6 (2.0)	4.5 (2.0)	−1.1 (2.5)	4.7 (2.4)	4.6 (2.8)	0.3 (3.4)	−1.4 (−3.5 to 0.7)
Pain on average in last 24 h	6.6 (2.0)	3.7 (1.8)	−2.8 (2.5)	5.9 (2.4)	3.7 (2.3)	−2.1 (3.1)	−0.7 (−2.7 to 1.3)
Pain on average in last week	5.9 (1.7)	4.2 (2.0)	−1.6 (2.5)	5.8 (1.9)	3.9 (2.0)	−1.6 (2.3)	0.0 (−1.7 to 1.8)
Pain on average in last month	6.0 (1.6)	4.3 (1.9)	−1.6 (2.0)	6.0 (1.9)	4.5 (1.9)	−1.2 (1.1)	−0.4 (−1.6 to 0.8)
Pain while walking in last week	6.5 (1.4)	4.3 (2.1)	−2.1 (2.4)	6.1 (2.1)	4.1 (2.1)	−1.7 (2.2)	−0.3 (−2.0 to 1.3)

Values are reported as mean (SD) unless otherwise stated

^a^Difference between outcomes at baseline and 12 weeks with missing values removed

#### Biomechanical outcome measures

Both groups demonstrated increased force under the midfoot when wearing their respective orthoses compared to the shoe-only condition (FFO = mean change 10.7 % BW [SD 6.6 %]; sham = mean change 4.4 % BW [SD 6.3 %]), yielding a group mean difference of 6.3 % BW (95 % CI 1.7 to 10.9). Evaluation of the peak hindfoot kinematics demonstrated that the FFO inverted the hindfoot relative to the shoe-only condition (mean = 0.7°, 95 % CI −0.1° to 1.5°), whereas the sham device everted the foot more (mean = −0.3°, 95 % CI −1.7° to +1.0°), yielding a group mean difference of 1.0° (95 % CI −0.5° to 2.6°).

## Discussion

The present study aimed to examine the role of a commercially available and commonly used treatment for midfoot OA and to assess whether a fully powered RCT is feasible. A future RCT powered to fully evaluate the effectiveness of FFOs in treating painful midfoot OA appears to be achievable based on observed recruitment, adherence, retention, blinding and the ability to detect small clinical differences between the orthoses intervention and sham groups.

### Feasibility outcomes

In physical devices, trials adherence in different treatment arms may explain clinical response. After 12 weeks, those in the sham group showed greater adherence than the FFO group, although a high proportion (93 % sham, 78 % FFO) of both met the minimal predefined adherence threshold. The difference may be due to the immediate comfort of the thinner sham device and the ability to accommodate them within in a wider variety of footwear (including slippers), compared to the FFO, which may have been less comfortable and more difficult to accommodate in a variety of shoes.

Blinding in physical device trials is rarely evaluated despite the potential visible differences between the intervention and placebo/sham devices [20]. In this study of participants who were naïve to orthoses, only one quarter of the group correctly identified the device, suggesting that blinding can be achieved where care is taken to ensure that devices are similar in appearance.

Recruitment took place over a pre-planned 8-month period, where an average of 15 potential participants were screened and 4.6 participants were recruited per month. Recruitment for a larger RCT would be feasible using a conservative estimate of 3.5 patients per month per centre (a 25 % reduction), employing a longer recruitment window and multiple recruiting centres.

### Clinical outcomes

In this feasibility study, a double-blind randomised controlled protocol was employed to minimise the influence of confounding factors and systematic bias. There was a trend towards the FFO group reporting a small improvement in pain and function compared to the sham group. These improvements were slightly smaller than our predefined minimal for clinically worthwhile improvement in pain (NRS = 1.5 point reduction) and function (MFPDI = ≥3 point reduction). Notably, while the FFO group demonstrated a 3.6 point reduction in subjective function (MFPDI) which exceeded the predefined minimally important difference, the sham group also demonstrated a 2.2-point reduction. A greater number of participants (36 %) in the FFO group reported improvement (using the PGIC scale) compared to the sham group. With improvements reported in both treatment arms, detection of placebo or natural history effects could only have been differentiated from the treatment effect by including a no-treatment arm. Overall, however, the reported improvements in participants’ pain, function and PGIC do support the hypothesis that the FFO may provide short-term clinical benefits. The clinical findings in this study are also consistent with previous studies examining the effect of foot orthoses on pain and function in midfoot OA patients [12, 13]. Taken together, these findings suggest that powered RCTs are now required.

The clinical improvements in both the FFO and sham groups are consistent with previous RCTs [30–35], suggesting there may be treatment benefit with some sham interventions due to the materials and manufacturing used not being entirely inert mechanically and therefore mediating foot pressures [20]. The specific sham intervention used in this study had minimal effect on midfoot forces and hindfoot kinematics and appears to be an adequate mechanical control for a definitive study but the interaction warrants exploration in a definitive RCT.

### Biomechanical outcome measures

To explore the effect of the different orthoses treatments in this study, a discrete number of biomechanical outcomes were investigated after 12 weeks of treatment to allow for acclimatisation and clinical use. The FFO intervention inverted the hindfoot by a small amount, whereas the sham allowed the hindfoot to evert by a similar magnitude. These findings are consistent with previous research that reported reduced hindfoot eversion when walking with three-quarter length FFOs, compared to full length or no orthoses [12]. There was an apparent between-group difference in force at the midfoot, with the FFO group yielding double the increase in force compared to sham group. These findings are corroborated in the literature, which suggests that increased midfoot pressures [11] and forces [29, 36] are observed with FFOs. The current biomechanical findings indicate a trend towards a different biomechanical effect for the two orthoses, with the FFOs appearing to restrict hindfoot motion while supporting the midfoot.

### Limitations

We recognise a number of limitations. The baseline matching for BMI and gender was relatively weak, and a future, larger study will need to manage such balance through stratification in the randomisation. Second, differences in pain or related functional scores did not meet pre-specified minimally important differences, and ordinal scales (NRS and MFPDI) may not be adequately sensitive to detect change over time. Future studies should supplement these subjective measures with objective measures of function such as kinematics and force, as was done in the present study and should consider using novel approaches such as activity monitoring that may be more sensitive in detecting changes in impairment and pain related function.

## Conclusion

The NICE clinical OA guidelines [14] suggest the use of orthoses as an adjunct treatment, although there is a lack of RCT evidence for their use in painful foot OA. Our present study shows that conducting a large trial is feasible in terms of recruitment, blinding, adherence and treatment effect. This study provides some evidence on how to detect patient improvement and that there is a measureable clinical difference between the FFO and sham groups that justifies further investigation with a fully powered RCT. To examine whether some of the improvement demonstrated in the sham group was associated with natural improvement, we would recommend that the definitive trial include a third, active-monitoring arm with no planned treatment, in order to better understand the placebo effect. This feasibility study suggests that implementation of a definitive RCT to evaluate the effectiveness of FFO for painful midfoot OA is achievable.

## Electronic supplementary material

Below is the link to the electronic supplementary material.

ESM 1 (DOCX 30 kb)
