# Supplementary material for: Foot orthoses in the treatment of symptomatic midfoot osteoarthritis using clinical and biomechanical outcomes: a randomised feasibility study
Source: Clin Rheumatol. 2015 Apr 28;35:987–96. doi: 10.1007/s10067-015-2946-6 (PMC4819552; doi:10.1007/s10067-015-2946-6)
Supplement: Supplementary file 1 — (DOCX 30 kb) [file 10067_2015_2946_MOESM1_ESM.docx]

Supplementary File 1 – Foot Orthoses Prescription

*Rationale for orthoses*

The functional foot orthoses VectOrthotic^®^ ([Healthy Step [Sensograph] Ltd](http://www.healthystep.co.uk/)) were chosen as the biomechanical “active” intervention for the OFFLOAD feasibility study. VectOrthotic^®^ is a commonly used pre-fabricated, modifiable device. This device is available in 50% of all national health services across the UK. This device was selected after meeting the following, previously described characteristics [1]: i) a rigid or semi-rigid shell, with a contoured medial arch, ii) a cupped heel, to control heel motion and iii) modifiable, to allow the addition of wedging or “posting” intended to influence the angular position of the device under the heel and midfoot. Pre-fabricated foot orthoses that met these characteristics were agreed by the study team as an optimal intervention. Most other pre-fabricated brands on the market in the UK (at the time of the study circa 2012-2013) did not have these characteristics. Using a pre-fabricated and semi-customised device allowed for fitting on the same day to reflect current clinical practice. Further work to establish the biomechanical role of customised foot orthoses was beyond the remit of this study.

VectOrthotic^®^ ([Healthy Step [Sensograph] Ltd](http://www.healthystep.co.uk/)) is a three quarter length polypropylene composite shell with a 4mm of uniform contoured compressed closed cell polyethylene cover and a range of optional rearfoot wedges (in sizes 2°, 4° and 6°) and optional VectOrthotic^®^ Extra cover made from a variable depth of polyethylene (6mm to 14mm) specifically designed to add midfoot support across the entire width.

In this study the VectOrthotic^®^ orthoses were, as per standard clinical practice, customised to the participant’s individual requirements and modified by an experienced clinical podiatric researcher (JH). Using the modification options included in the VectOrthotic^®^ pack, the customisation process included the possibility of applying a range of medial rear foot wedges (in sizes 2°, 4° and 6°) and choosing between two compressed closed cell polyethylene covers; standard uniform cover or the ‘VectOrthotic^®^ Extra cover (with midfoot support)

*Clinical assessment*

The application of rear foot wedges and cushioned cover was based on standing foot posture and the height of the navicular in both the neutral and relaxed standing foot posture positions. The navicular height was chosen to represent the midfoot as it is used as a clinical tool and the static movement from a neutral to relaxed standing posture can over-estimate the motion of the midfoot in walking and running motion [2-4]. This would suggest that posting the orthoses to the height of the navicular in neutral posture would support the midfoot through the stance phase of gait [4].

*Customisation of FFO*

Orthoses modifications were made in a stepwise approach starting from the standard top cover and basic shell, adding wedges and then altering the top cover accordingly. Firstly navicular drop was visually inspected barefoot [4]. The three quarter length rigid shell and 4mm standard cover was placed beneath the feet and the clinical researcher (JH) placed the participant in a neutral standing posture [2-3]. The height of the navicular and the position of the arch relative to the orthotic device were visually assessed to ensure the surfaces were congruent. The patient was then asked to relax their foot posture. The orthotic device and the modifications were deemed to be appropriate if the arch remained supported as shown by a visual reduction in navicular drop, compared to the previous barefoot inspection. If there was no arch congruence or midfoot support (as evidenced by visualising the navicular drop) then wedges were added in a systematic nature; increasing the arch height by 2°, 4° and 6° and the Extra cover was added to aid congruence and support. For instance, for those with a higher arch profile, a 6° medial heel wedge and the VectOrthotic^®^ Extra cover was added.

Finally, the length of the top cover was fitted to the length of inside of the shoe, with advice to trim the length if the orthoses were required to fit other shoes.

*Sham Intervention*

The sham intervention group received orthoses that mimicked the appearance of the active intervention. This was achieved by provided only the VectOrthotic^®^ top cover (4mm depth of uniform contoured compressed closed cell polyethylene). This was fitted to the length of the shoe, with advice to reduce the length of the orthoses if required to fit other shoes.

All participants were provided with an information leaflet about the fitting of an orthoses within the shoe. They were recommended to contact the researcher if the orthoses caused rubbing or new pains.

**References**
1. Redmond A, Lumb P, Landorf K. Effect of cast and noncast foot orthoses on plantar pressure and force during normal gait. Journal of the American Podiatric Medical Association 2000;90(9):441-9.

2. Evans AM, Copper AW, Scharfbillig RW, Scutter SD, Williams MT. Reliability of the foot posture index and traditional measures of foot position. Journal of the American Podiatric Medical Association. 2003;93(3):203-13.

3. Redmond AC(1), Crosbie J, Ouvrier RA. Development and validation of a novel rating system for scoring standing foot posture: the Foot Posture Index. Clinical Biomechanics 2006; 21(1):89-98.

4. Dicharry JM, Franz JR, Della Croce U, Wilder RP, Riley PO, Kerrigan DC. Differences in static and dynamic measures in evaluation of talonavicular mobility in gait. Journal of Orthopaedic & Sports Physical Therapy. 2009;39(8):628-34.
